# Secure Degrees of Freedom in Networks with User Misbehavior

**DOI:** 10.3390/e21100945

**Published:** 2019-09-26

**Authors:** Karim Banawan, Sennur Ulukus

**Affiliations:** 1 Faculty of Engineering, Alexandria University, Alexandria 21544, Egypt; 2 Department of ECE, University of Maryland, College Park, MD 20742, USA

**Keywords:** secure degrees of freedom, interference alignment, extensive-form games

## Abstract

We investigate the secure degrees of freedom (s.d.o.f.) of three new channel models: broadcast channel with combating helpers, interference channel with selfish users, and multiple access wiretap channel with deviating users. The goal of introducing these channel models is to investigate various malicious interactions that arise in networks, including active adversaries. That is in contrast with the common assumption in the literature that the users follow a certain protocol altruistically and transmit both message-carrying and cooperative jamming signals in an optimum manner. In the first model, over a classical broadcast channel with confidential messages (BCCM), there are two helpers, each associated with one of the receivers. In the second model, over a classical interference channel with confidential messages (ICCM), there is a helper and users are selfish. By casting each problem as an extensive-form game and applying recursive real interference alignment, we show that, for the first model, the combating intentions of the helpers are neutralized and the full s.d.o.f. is retained; for the second model, selfishness precludes secure communication and no s.d.o.f. is achieved. In the third model, we consider the multiple access wiretap channel (MAC-WTC), where multiple legitimate users wish to have secure communication with a legitimate receiver in the presence of an eavesdropper. We consider the case when a subset of users deviate from the optimum protocol that attains the exact s.d.o.f. of this channel. We consider two kinds of deviation: when some of the users stop transmitting cooperative jamming signals, and when a user starts sending intentional jamming signals. For the first scenario, we investigate possible responses of the remaining users to counteract such deviation. For the second scenario, we use an extensive-form game formulation for the interactions of the deviating and well-behaving users. We prove that a deviating user can drive the s.d.o.f. to zero; however, the remaining users can exploit its intentional jamming signals as cooperative jamming signals against the eavesdropper and achieve an optimum s.d.o.f.

## 1. Introduction

Physical layer security techniques allow secure transmission of information (in absolute sense) without the need for encryption keys [1]. Consequently, the problems of exchanging encryption keys across open wireless networks are mitigated. In the seminal work [2], Wyner showed that secure communication through a degraded wiretap channel is possible by exploiting the noisy nature of the channel. The problem was extended to general wiretap channel, which may not be necessarily degraded by Csiszar and Korner in [3]. The physical layer security framework was then extended to various multiuser settings such as: the multiple access wiretap channel (MAC-WTC) [4], broadcast channel with confidential messages (BCCM) [5,6,7,8,9], interference channel with confidential messages (ICCM) [5], multireceiver wiretap channels [10,11], and relay-eavesdropper channels [12]. In the absence of exact secrecy rates, secure degrees of freedom (s.d.o.f.) provide a first order approximation to the secrecy rate by giving their scaling with $\frac{1}{2}\log P$, where *P* is the total average transmitted power. The s.d.o.f. have been considered in the literature in many multiuser channel models, such as helper wiretap channel [13,14], multiple-access wiretap channel [13,15,16,17], interference channel [13,17,18,19,20,21,22], *X*-channel [23,24], half-duplex relay channel [25], compound wiretap channel [26], diamond channel [27], MIMO wiretap *Y* channel [28], multiuser channel models under imperfect CSIT [29,30,31,32,33]. An investigation of the intercept probability in the presence of eavesdropping attack and interference can be found in [34].

In this work, we investigate extended versions of BCCM, ICCM, and MAC-WTC channel models. Information-theoretic security for discrete memoryless interference and broadcast channels with confidential messages were studied in [5]. BCCM consists of a transmitter and two receivers. The transmitter has two messages, each directed to one of the receivers and needing to be kept secure from the other receiver. The s.d.o.f. of Gaussian BCCM is zero for each user [13]. However, with an altruistic system helper, each user in the BCCM can have an s.d.o.f. of 1/2 [13]. ICCM consists of two transmitters and two receivers. Each transmitter has a message that needs to be conveyed reliably to one of the receivers and needs to be kept secret from the other receiver. The s.d.o.f. of Gaussian ICCM is 1/3 for each user [13]. With an altruistic system helper, each user in the ICCM can have an s.d.o.f. of 1/2 [13]. In both of these systems, this eventual 1/2 s.d.o.f. per user requires perfect coordination between the transmitters and the helper, even if that obliges the transmitters to jam their own receivers as in the case of ICCM.

In MAC-WTC, which was introduced in [4,35], multiple legitimate users wish to have secure communication with a legitimate receiver in the presence of an eavesdropper. The secrecy capacity region of the MAC-WTC is still unknown, even in the simple Gaussian setting [4,13,15,17,35,36,37]. Recently, [13] and [17] determined the *exact* sum s.d.o.f. and the entire s.d.o.f. region, respectively, of the MAC-WTC. The exact sum s.d.o.f. of a *K*-user MAC-WTC is $\frac{K(K-1)}{K(K-1)+1}$ [13]. The achievability of this sum s.d.o.f. requires all users to send signals in a certain optimum manner. The main tools in the achievability are: structured signaling, channel prefixing, cooperative jamming, and interference alignment. In the optimum scheme, each user sends K−1 streams of message-carrying signals and 1 stream of cooperative jamming signal. The signals are simultaneously aligned at the two receivers: At the eavesdropper, all message-carrying signals are aligned with a cooperative jamming signal, which ensures that the information leakage to the eavesdropper is zero in the s.d.o.f. sense; at the legitimate receiver, all cooperative jamming signals are aligned in a single dimension occupying the smallest space, thereby leaving the largest space for message-carrying signals. The total number of dimensions created at the legitimate receiver is K(K−1)+1, and one dimension is lost for the cooperative jamming signals, hence achieving a sum s.d.o.f. of $\frac{K(K-1)}{K(K-1)+1}$.

All these works assume that all nodes are *altruistic* and follow a prescribed transmission policy in order to maximize the sum secure rate of the entire system. In this paper, we investigate BCCM, ICCM, and MAC-WTC channel models in the case of selfish and malicious behavior, where the users/helpers do not perform the system-wide-optimal altruistic behavior but apply a selfish strategy and/or take sides by aiming to help one user and potentially hurt the other. These new models are extensions of the ones studied in [4,5,13] and are a step forward in studying channel models with active adversaries. We use s.d.o.f. metric to quantify the effects of these malicious behaviors. For BCCM and ICCM channel models, we note a self-enforcing property: Even with the excessive capabilities of the helpers/users (infinite power and all-knowing entities), these capabilities are naturally restricted in these channel models due to the users’/helpers’ interest in reliable communication to/with their own receivers. That is, no entity can use infinite powered Gaussian jamming signals which would wipe out the communication for everybody. This self-enforcing property necessitates users to apply selective jamming via interference alignment. This motivates studying such jamming techniques and analyzing their effect on the s.d.o.f. of the users. In addition, a careful look at the achievable scheme for the MAC-WTC in [13] reveals that the cooperative jamming signal of each user protects parts of the message-carrying signals of the other users; and that no user can protect its own signals. This creates an interesting ecosystem where each user strictly depends on the rest of the users for its own security. The fact that a user’s cooperative jamming transmission does not contribute to its own security but at the same time uses up its own transmit power may motivate some selfish users not to send cooperative jamming signals. In this work, we investigate the effects of such (and worse) deviations from the optimum signaling scheme on the system s.d.o.f., and the actions that the rest of the users can take to compensate for such behavior.

In the first model, which is the *BCCM with combating helpers*, there are two helpers, where each helper takes the side of one of the receivers and at the same time aims to hurt the secure communication to the other receiver. The two helpers have contradicting objectives and hence are combating. Helpers in this model do not coordinate with the transmitter as in [13]. We use a stringent objective function for each helper: Each helper minimizes the s.d.o.f. of the other receiver, while not decreasing the s.d.o.f. of its own receiver by its action. We formulate the problem as an extensive-form game [38], which is a sequential strategic game, where every player (node) acts according to its information about the other nodes’ actions in previous transmission frames. We investigate achievable schemes that use real interference alignment [39] in a recursive way. We prove that under this stringent objective function and recursive real interference alignment, the malicious behaviors of the two combating helpers are neutralized, and the s.d.o.f. for each user converges to the optimal s.d.o.f. of 1/2 per user [13], as if both helpers are altruistic.

In the second model, which is the *ICCM with selfish users*, there is an external system helper. In this model, the users do not coordinate as in the optimal strategy in [13] instructs. The users are selfish and want to hurt the other receiver; each transmitter’s goal is to maximize the difference of the s.d.o.f. between the two receivers. This permits each user to jam its own receiver if this hurts the other receiver more, making self-jamming more natural here than the optimum scheme in [13]. There is a neutral helper in this system which aims to maximize the s.d.o.f. of the system. Using the extensive-form game formulation and recursive real interference alignment, we show that the selfishness of the users precludes any secure communication and drives the s.d.o.f. of both users to zero, despite the existence of a mediating helper.

In the third model, which is the *MAC-WTC with deviating users*, we first consider the case where *M* out of *K* users deviate by not transmitting cooperative jamming signals. We start by evaluating the achievable sum s.d.o.f. when the remaining users do not change their original optimum strategies. We show that the sum s.d.o.f. of the system decreases, and deviating users do not benefit from their actions. Then, we consider two possible counterstrategies by the remaining users: In the first strategy, all users decrease their rates to ensure that all message-carrying signals are protected by the remaining cooperative jamming signals, and leakage s.d.o.f. is zero. We show that, in this case, the individual s.d.o.f. of the deviating users increase. Hence, deviating users gain at the expense of well-behaving users. In the second strategy, we allow the leakage s.d.o.f. to be nonzero but constrain leakage in a single dimension. We show that, although the sum s.d.o.f. of the system is lower than in the case of the first counterstrategy, this strategy decreases the individual s.d.o.f. of the deviating users and increases the s.d.o.f. of well-behaving users. Next, we consider a more severe form of deviation by considering one user turning malicious and sending intentional jamming signals. As this deviating user has infinite power, it can wipe out all communication, secure or otherwise, if it sends Gaussian signals. For the sake of a meaningful formulation, we restrict the strategy set of this deviating user to be of structured signaling and alignment type. Under this restriction, we formulate the problem as an extensive-form game [38]. We show that this deviating user can drive the s.d.o.f. of the system to zero. We then show that, interestingly, the remaining users can utilize these intentional (malicious) jamming signals to protect more message-carrying signals at the eavesdropper, achieving a sum s.d.o.f. of $\frac{{\left(K-1\right)}^{2}}{{\left(K-1\right)}^{2}+1}$. We prove that this sum s.d.o.f. matches the sum s.d.o.f. of a K−1 user MAC-WTC with 1 external altruistic helper, thereby showing that the system turns a malicious jammer into an altruistic helper, i.e., the deviating user benefits the system against its intentions.

*Organization:* In Section 2, we focus on the BCCM with combating helpers. In Section 3, we consider the ICCM with selfish users. Finally, in Section 4, we consider the MAC-WTC with deviating users. For each model, we first give the formal description of the channel model, then we present our proposed achievable schemes.

## 2. BCCM with Combating Helpers

### 2.1. System Model and Assumptions

In BCCM, the transmitter has two private messages $W_{1}$ and $W_{2}$ picked from the message sets $W_{1},W_{2}$ uniformly with rates $R_{1}$, $R_{2}$, respectively, where $R_{i}=\frac{1}{n}\log\left|W_{i}\right|$, where *n* is the length of the codeword. Each message $W_{i}$ should be received reliably by the *i*th receiver, while being kept secure from the *j*th receiver, i≠j:

(1) $$\begin{array}{cc} P({\hat{W}}_{1}\neq W_{1}) & \leq \epsilon ,\;P({\hat{W}}_{2}\neq W_{2})\leq \epsilon \end{array}$$

(2) $$\begin{array}{cc} \frac{1}{n}I\left(W_{2};Y_{1}^{n}\right) & \leq \epsilon ,\;\frac{1}{n}I\left(W_{1};Y_{2}^{n}\right)\leq \epsilon \end{array}$$

where I(X;Y) is the mutual information between the random variables, *X* and *Y*, and ${\hat{W}}_{i}$ is the estimate of $W_{i}$ at the *i*th receiver. The s.d.o.f. $d_{i}$ is defined as $d_{i}=\lim_{P\to \infty }\frac{R_{i}}{\frac{1}{2}\log P}$, where *P* is the transmitter power constraint $E[X^{2}]\leq P$.

**Figure 1:**
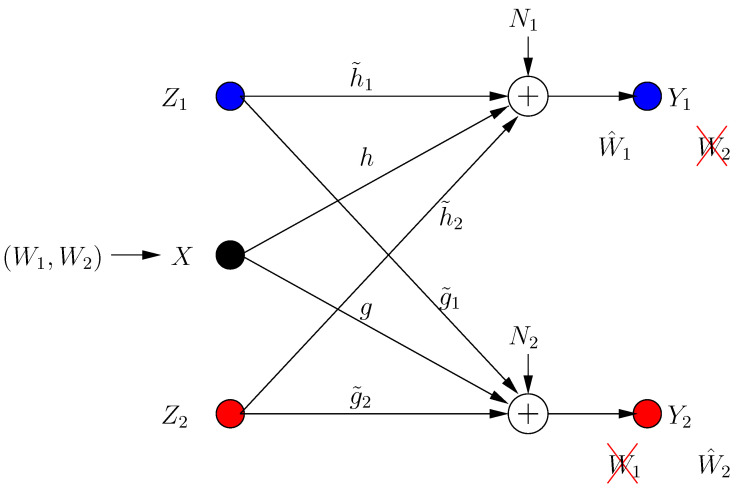
Broadcast channel with confidential messages (BCCM) with combating helpers.

The system has two helpers with inputs $Z_{1}$ and $Z_{2}$, with the power constraints $E[Z_{i}^{2}]\leq P$. Each helper assists secure transmission to *one* of the receivers. The input/output relations for the BCCM with combating helpers (see Figure 1) are:

(3) $$\begin{array}{cc} Y_{1}\left[k\right] & =hX\left[k\right]+{\tilde{h}}_{1}Z_{1}\left[k\right]+{\tilde{h}}_{2}Z_{2}\left[k\right]+N_{1}\left[k\right] \end{array}$$

(4) $$\begin{array}{cc} Y_{2}\left[k\right] & =gX\left[k\right]+{\tilde{g}}_{1}Z_{1}\left[k\right]+{\tilde{g}}_{2}Z_{2}\left[k\right]+N_{2}\left[k\right] \end{array}$$

where $Y_{i}\left[k\right]$ is the received signal at the *i*th receiver in the *k*th transmission frame, *h*, *g* are the channel gains from the transmitter to receivers 1, 2, respectively, and ${\tilde{h}}_{i}$, ${\tilde{g}}_{i}$ are the channel gains from helper *i* to receivers 1, 2, respectively.

The helpers are *combating* as they maximize the s.d.o.f. of one user only, while hurting the other user by sending jamming signals. The transmitter acts in even transmission frames, and helpers respond in odd frames. Each node has perfect channel state information (CSI) and knows the actions of others at the end of every frame. We require that the action of a helper does not hurt its own receiver (in terms of s.d.o.f.) if no new jamming signals are produced by the other helper. Consequently, we formalize the role of the *i*th helper as:

(5) $$\begin{array}{c} \min\;d_{j}\left(k\right)\;s.t.\;d_{i}\left(k\right)=d_{i}\left(k-1\right) \end{array}$$

where i,j∈{1,2},i≠j and $d_{j}\left(k\right)$ is the s.d.o.f. of the *j*th user in the *k*th transmission frame, where *k* is odd. On the other hand, the transmitter does not take the side of any of the users and maximizes the sum s.d.o.f. of the system, i.e., transmitter’s role in even encoding frames is:

(6) $$\max\;d_{1}\left(k\right)+d_{2}\left(k\right)$$

### 2.2. Achievable Scheme: Recursive Real Interference Alignment as Extensive-Form Game

We use recursive real interference alignment as the achievable strategy for our model. At encoding frame *k*, all secure and jamming signals are picked from PAM constellation set $C(a_{k},Q_{k})$, where $a_{k}$ is the minimum distance between any two points in the constellation and $Q_{k}$ is the number of points.

#### 2.2.1. For Frames k=0, k=1

Frames 0 and 1 are considered transient frames. For frame 0, the transmitter performs the optimal strategy in the presence of helpers [13] and sends two signal components $V_{11}$, $V_{21}$ in two irrational dimensions:

(7) $$X\left[0\right]=\alpha_{1}V_{11}+\alpha_{2}V_{21}$$

where $\alpha_{1}$, $\alpha_{2}$ are rationally independent scalars. These message-carrying signals are not secured. None of the helpers expects the other helper to jam its own receiver; thus, each helper needs to protect the message of its own receiver at the other receiver. Hence, at k=1, the *i*th helper sends a structured jamming signal ${\tilde{U}}_{i1}$ in the irrational dimension where its message-carrying signal lies at the other receiver as:

(8) $$\begin{array}{c} Z_{1}\left[1\right]=\frac{\alpha_{1}g}{{\tilde{g}}_{1}}{\tilde{U}}_{11},\;Z_{2}\left[1\right]=\frac{\alpha_{2}h}{{\tilde{h}}_{2}}{\tilde{U}}_{21} \end{array}$$

Then, the received signals are:

(9) $$\begin{array}{cc} Y_{1}\left[1\right] & =\alpha_{1}hV_{11}+\frac{\alpha_{1}g{\tilde{h}}_{1}}{{\tilde{g}}_{1}}{\tilde{U}}_{11}+\alpha_{2}h\left(V_{21}+{\tilde{U}}_{21}\right)+N_{1} \end{array}$$

(10) $$\begin{array}{cc} Y_{2}\left[1\right] & =\alpha_{2}gV_{21}+\frac{\alpha_{2}h{\tilde{g}}_{2}}{{\tilde{h}}_{2}}{\tilde{U}}_{21}+\alpha_{1}g\left(V_{11}+{\tilde{U}}_{11}\right)+N_{2} \end{array}$$

**Figure 2:**
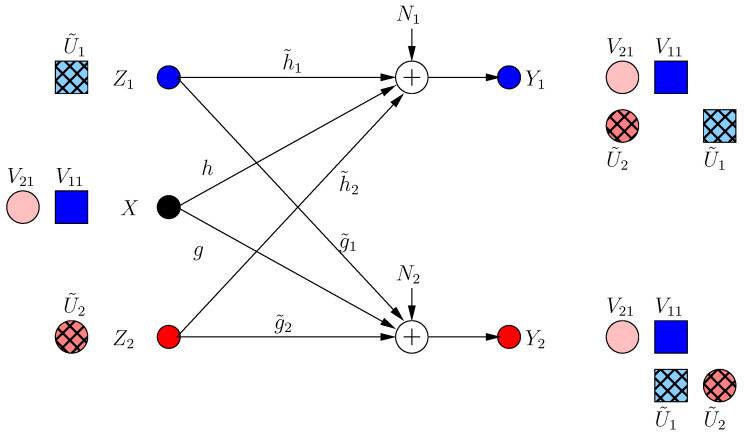
BCCM frame k=1. Pink circle and blue square denote user signals, and the hatched circles/squares denote corresponding helper jamming signals.

Although $V_{11}$, $V_{21}$ are now secure, this results in a new irrational dimension at each receiver as in Figure 2. Hence, $d_{i}\left(1\right)=1/3$ for each user as we show formally in Section 2.3 (instead of $d_{i}=1/2$ in BCCM with coordinating helpers).

#### 2.2.2. For Frame k=2

The transmitter knows that a new irrational dimension is generated within frame k=1. The transmitter uses this dimension in its favor, as it can protect more message-carrying signals. It produces two new message-carrying signal components $V_{12}$, $V_{22}$ to be aligned with the generated jamming dimensions in frame k=1 as:

(11) $$\begin{array}{cc} X[2] & =\alpha_{1}V_{11}+\alpha_{2}V_{21}+\frac{\alpha_{2}h{\tilde{g}}_{2}}{{\tilde{h}}_{2}g}V_{12}+\frac{\alpha_{1}g{\tilde{h}}_{1}}{{\tilde{g}}_{1}h}V_{22} \end{array}$$

(12) $$\begin{array}{cc} \; & =X\left[1\right]+\beta_{1}V_{12}+\beta_{2}V_{22} \end{array}$$

**Figure 3:**
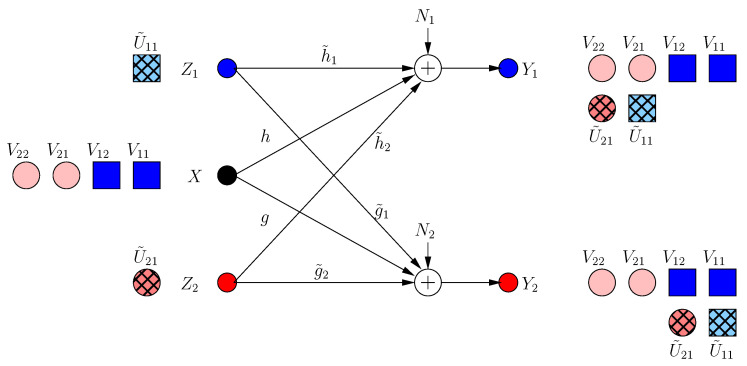
BCCM frame k=2.

That is, the transmitter appends its last frame transmission with two new signal components in rationally independent dimensions $\beta_{1}$, $\beta_{2}$ (see Figure 3). The received signals are:

(13) $$\begin{array}{cc} Y_{1}\left[1\right]= & \alpha_{1}hV_{11}+\frac{\alpha_{2}h^{2}{\tilde{g}}_{2}}{{\tilde{h}}_{2}g}V_{12}+\frac{\alpha_{1}g{\tilde{h}}_{1}}{{\tilde{g}}_{1}}\left(V_{22}+{\tilde{U}}_{11}\right)+\alpha_{2}h\left(V_{21}+{\tilde{U}}_{21}\right)+N_{1} \end{array}$$

(14) $$\begin{array}{cc} Y_{2}\left[1\right]= & \alpha_{2}gV_{21}+\frac{\alpha_{1}g^{2}{\tilde{h}}_{1}}{{\tilde{g}}_{1}h}V_{22}+\frac{\alpha_{2}h{\tilde{g}}_{2}}{{\tilde{h}}_{2}}\left(V_{12}+{\tilde{U}}_{12}\right)+\alpha_{1}g\left(V_{11}+{\tilde{U}}_{11}\right)+N_{2} \end{array}$$

Consequently, the system retains full s.d.o.f. ($d_{i}\left(2\right)=1/2$).

#### 2.2.3. For Frame k=3

Now, each helper minimizes the s.d.o.f. of the other user by sending a jamming signal. However, due to the strong constraint $d_{i}\left(3\right)=d_{i}\left(2\right)$, no helper jams the other receiver directly, as this would create a new jamming dimension at the side of its own receiver, decreasing its own s.d.o.f. Instead, it transmits a jamming signal which aligns with the already jammed dimension at its own receiver as:

(15) $$\begin{array}{c} Z_{1}\left[3\right]=Z_{1}\left[1\right]+\frac{\alpha_{2}h}{{\tilde{h}}_{1}}{\tilde{U}}_{12},\;Z_{2}\left[3\right]=Z_{2}\left[1\right]+\frac{\alpha_{1}g}{{\tilde{g}}_{2}}{\tilde{U}}_{22} \end{array}$$

Consequently, the received signals are:

(16) $$\begin{array}{cc} Y_{1}\left[3\right] & =Y_{1}\left[2\right]+\alpha_{2}h{\tilde{U}}_{12}+\frac{\alpha_{1}{\tilde{h}}_{2}g}{{\tilde{g}}_{2}}{\tilde{U}}_{22} \end{array}$$

(17) $$\begin{array}{cc} Y_{2}\left[3\right] & =Y_{2}\left[2\right]+\alpha_{1}g{\tilde{U}}_{22}+\frac{\alpha_{2}{\tilde{g}}_{2}h}{{\tilde{h}}_{1}}{\tilde{U}}_{12} \end{array}$$

**Figure 4:**
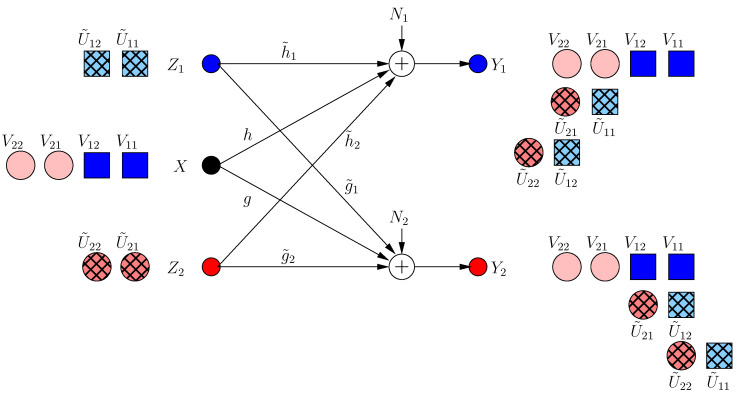
BCCM frame k=3.

Since the $\alpha_{2}h$ dimension is already jammed, the first helper does not create a new irrational dimension. Hence, it does not hurt its own receiver. However, it creates a new jamming dimension $\frac{\alpha_{2}{\tilde{g}}_{2}h}{{\tilde{h}}_{1}}$ at the second receiver, which decreases the resultant s.d.o.f. From the symmetry, the second helper applies the same strategy, and hence, the resulting s.d.o.f. is $d_{i}\left(3\right)=2/5$ as in Figure 4. Note that neither of the helpers can hold back its original jamming signal (i.e., each helper should append its previous signaling with new jamming signals), because if not, its previous message-carrying signals are compromised.

#### 2.2.4. For General *k*th Frame

If *k* is odd, the helpers produce one extra jamming component aligned with the last generated jamming signal of the other helper. If *k* is even, the transmitter makes use of this jamming signal and provides two extra secure signals, achieving the maximum possible s.d.o.f. ($d_{i}\left(k\right)=1/2$, *k* is even).

### 2.3. Calculation of the Secure Degrees of Freedom

To calculate the s.d.o.f., we need the following lemma.

**Lemma** **1.** *If every message-carrying signal is protected by a cooperative jamming signal, then the s.d.o.f. is given by:*

(18) $$d_{i}\left(k\right)=\frac{J_{k}}{L_{k}}$$

*where $J_{k}$ is the number of irrational dimensions needed to receive the message-carrying signal of user i at the kth frame $V_{i}\left[k\right]={\left[V_{i1},V_{i2},\cdots V_{iJ_{k}}\right]}^{T}$ and $L_{k}$ is the total number of irrational dimensions.*

**Proof.**  From [5], the following rate expression is achievable for the BCCM:

(19) $$\begin{array}{cc} R_{1}\left[k\right] & \geq I\left(V_{1}\left[k\right];Y_{1}\left[k\right]\right)-I\left(V_{1}\left[k\right];Y_{2}\left[k\right]|V_{2}\left[k\right]\right) \end{array}$$

Let $L_{k}$ denote the total number of irrational dimensions used in the *k*th frame at receiver 1, and $J_{k}$ denote the number of dimensions used to receive $V_{1}\left[k\right]$ at receiver 1 (without loss of generality, due to symmetry). Then, by choosing $Q_{k}=P^{\frac{1-\delta }{2(L_{k}+\delta )}}$ and $a_{k}=\gamma P^{\frac{1}{2}}/Q_{k}$, the average power constraint is satisfied for all nodes, and the probability of error is upper bounded using the Khintchine–Groshev theorem of Diophantine approximation in number theory as in [39] as:

(20) $$P\left({\hat{V}}_{i}\left[k\right]\neq V_{i}\left[k\right]\right)\leq \exp\left(-\eta_{\gamma }P^{\delta }\right)$$

where $\eta_{\gamma }$ is constant that does not depend on *P*. Hence, the probability of error converges to zero as P→∞. Then, using Fano’s inequality and the data processing inequality of $V_{i}\left[k\right]\to Y_{i}\left[k\right]\to {\hat{V}}_{i}\left[k\right]$, we lower bound $I(V_{i};Y_{i}\left[k\right])$ as follows:

(21) $$\begin{array}{cc} I(V_{i}\left[k\right];Y_{i}\left[k\right]) & =H\left(V_{i}\left[k\right]\right)-H\left(V_{i}\left[k\right]|Y_{i}\left[k\right]\right) \end{array}$$

(22) $$\begin{array}{cc} \; & \geq H\left(V_{i}\left[k\right]\right)-H\left(V_{i}\left[k\right]|{\hat{V}}_{i}\left[k\right]\right) \end{array}$$

(23) $$\begin{array}{cc} \; & \geq \left[1-\exp\left(-\eta_{\gamma }P^{\delta }\right)\right]\log{\left(2Q+1\right)}^{J_{k}}-1 \end{array}$$

(24) $$\begin{array}{cc} \; & =\frac{J_{k}\left(1-\delta \right)}{L_{k}+\delta }\left(\frac{1}{2}\log P\right)+o\left(\log P\right) \end{array}$$

Since we designed the coding scheme at each frame so that $V_{1}\left[k\right]$ is completely hidden for some $U_{1}\left[k\right]$, we can upper bound the second term as:

(25) $$\begin{array}{cc} I(V_{1}\left[k\right];Y_{2}\left[k\right]|V_{2}\left[k\right]) & \leq I\left(V_{1}\left[k\right];A\left[k\right]\left(V_{1}\left[k\right]+U_{1}\left[k\right]\right)\right) \end{array}$$

(26) $$\begin{array}{cc} \; & =H\left(V_{1}\left[k\right]+U_{1}\left[k\right]\right)-H\left(U_{1}\left[k\right]\right) \end{array}$$

(27) $$\begin{array}{cc} \; & =\log{\left(4Q+1\right)}^{J_{k}}-\log{\left(2Q+1\right)}^{J_{k}} \end{array}$$

(28) $$\begin{array}{cc} \; & \leq J_{k} \end{array}$$

where A[k] is a diagonal matrix which corresponds to the irrational-dimension gains. The last step follows from carefully designing the jamming vector $U_{1}\left[k\right]$, so that it aligns with each component of $V_{1}\left[k\right]$. By taking limit as P→∞, we have $d_{i}\left(k\right)=\frac{J_{k}}{L_{k}}$. □

Now, we are ready to formally calculate the resulting s.d.o.f. from the recursive real interference alignment in the following theorem.

**Theorem** **1.** *For BCCM with combating helpers under the constraint of not decreasing the s.d.o.f. of their own receivers due to helper actions, the s.d.o.f. of each user evolves as:*

(29) $$d_{i}\left(k\right)=\left\{\begin{array}{cc} 1/2, & k\;even\\ \frac{k+1}{2k+4}\to 1/2, & k\;odd \end{array}\right.$$

*i.e., the combating behavior is asymptotically neutralized.*

**Proof.**  Using Lemma 1, we have $d_{i}\left(k\right)=\frac{J_{k}}{L_{k}}$. We complete the proof by calculating the dimensions $J_{k}$, $L_{k}$. We prove this by induction on *k*. For the base step k=1, we have $J_{k}=1$ and $L_{k}=3$ which conforms with (29). For k=2, we have $J_{k}=2$ and $L_{k}=4$, and hence, $d_{i}\left(k\right)=1/2$.

For the induction step, assume that *k* is odd and $d_{i}\left(k-2\right)=\frac{k-1}{2k}$. Then, in the (k−1)th frame, the transmitter can always add two extra message-carrying signals to have $d_{i}\left(k-1\right)=1/2$. Thus, $J_{k-1}=J_{k-2}+1$ and $L_{k-1}=L_{k-2}+1$. This is because the transmitter uses the extra irrational dimension produced by jamming in odd frames in its favor, hence adding one extra dimension corresponding to the new message-carrying signal. This results in the following simultaneous equations:

(30) $$\begin{array}{c} \frac{J_{k-2}}{L_{k-2}}=\frac{k-1}{2k},\;\frac{J_{k-1}}{L_{k-1}}=\frac{J_{k-2}+1}{L_{k-2}+1}=\frac{1}{2} \end{array}$$

Solving these two equations gives $L_{k-2}=k$ and $J_{k-2}=\frac{\left(k-1\right)}{2}$. Then, $L_{k-1}=k+1$ and $J_{k-1}=\frac{k+1}{2}$. In the next frame transmission, each helper produces an extra jamming component aligned with a already jammed dimension. This increases $L_{k}$ by one at the other receiver without changing $J_{k}$. Consequently, $d_{i}\left(k\right)=\frac{J_{k}}{L_{k}}=\frac{\frac{k+1}{2}}{k+2}=\frac{k+1}{2k+4}$, which converges to 1/2. □

## 3. ICCM with Selfish Users

### 3.1. System Model and Assumptions

**Figure 5:**
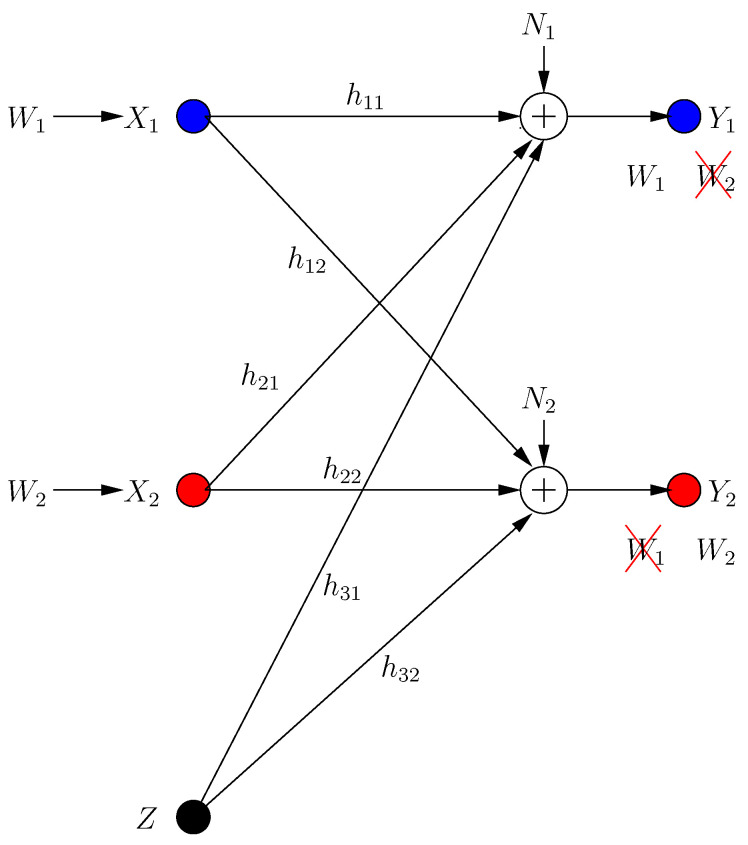
Interference channel with confidential messages (ICCM) with selfish users.

In ICCM, each transmitter has a message $W_{i}$ picked from the message set $W_{i}$ uniformly with rate $R_{i}=\frac{1}{n}\log\left|W_{i}\right|$ for i∈{1,2}. Message $W_{i}$ should be received reliably by the *i*th receiver, while being kept secure from the *j*th receiver, i≠j. The system has an external helper with channel input *Z*. Inputs satisfy power constraints $E[X_{i}^{2}]\leq P$ and $E[Z^{2}]\leq P$. The ICCM model depicted in Figure 5 is given by:

(31) $$\begin{array}{cc} Y_{1}\left[k\right] & =h_{11}X_{1}\left[k\right]+h_{21}X_{2}\left[k\right]+h_{31}Z\left[k\right]+N_{1}\left[k\right] \end{array}$$

(32) $$\begin{array}{cc} Y_{2}\left[k\right] & =h_{12}X_{1}\left[k\right]+h_{22}X_{2}\left[k\right]+h_{32}Z\left[k\right]+N_{2}\left[k\right] \end{array}$$

where $Y_{i}\left[k\right]$ is the received signal at the *i*th receiver in the *k*th transmission frame, and $h_{ij}$ is the channel gain from transmitter i=1,2,3 (transmitter 3 is the helper) to receiver j=1,2.

The users are *selfish* and malicious. User *i* maximizes the individual s.d.o.f. at receiver $Y_{i}$, while maximally hurting the second user. Formally, the *i*th user’s role is:

(33) $$\max\;d_{i}\left(k\right)-d_{j}\left(k\right)$$

where i≠j,i,j∈{1,2}. The role of the users here is *less stringent* than in the BCCM model, since in the ICCM model, we allow the users to hurt their own receivers if they hurt the other receiver more. On the other hand, the system helper does not take the side of any of the users and maximizes the sum s.d.o.f. of the system:

(34) $$\max\;d_{i}\left(k\right)+d_{j}\left(k\right)$$

### 3.2. Achievable Scheme: Recursive Real Interference Alignment as Extensive Form Game

Similar to the BCCM, we propose using recursive interference alignment using the PAM constellation $C(a_{k},Q_{k})$.

#### 3.2.1. For Frame k=0

All nodes perform the optimal *selfless* strategy as in [13]. The transmitted signals are:

(35) $$\begin{array}{c} X_{1}\left[0\right]=\frac{h_{32}}{h_{12}}V_{11},\;X_{2}\left[0\right]=\frac{h_{31}}{h_{21}}V_{21},\;Z\left[0\right]={\tilde{U}}_{1} \end{array}$$

**Figure 6:**
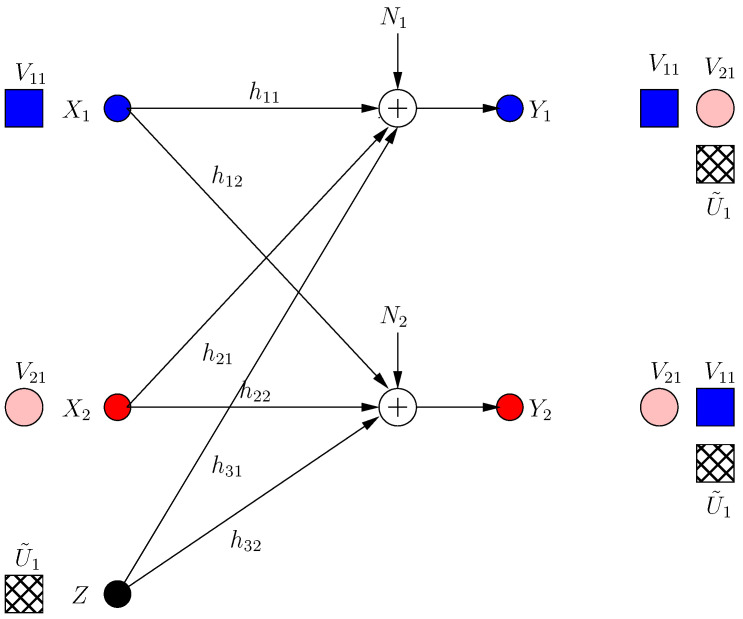
ICCM frame k=0. Pink circle and blue square denote user signals, and the hatched squares denote jamming signals.

The received signals at both receivers are (as in Figure 6):

(36) $$\begin{array}{cc} Y_{1}\left[0\right] & =\frac{h_{32}h_{11}}{h_{12}}V_{11}+h_{31}\left(V_{21}+{\tilde{U}}_{1}\right)+N_{1} \end{array}$$

(37) $$\begin{array}{cc} Y_{2}\left[0\right] & =\frac{h_{31}h_{22}}{h_{21}}V_{21}+h_{32}\left(V_{11}+{\tilde{U}}_{1}\right)+N_{2} \end{array}$$

which implies that the achievable s.d.o.f. $d_{i}\left(0\right)=1/2$.

#### 3.2.2. For Frame k=1

User *i* maximizes $d_{i}\left(1\right)-d_{j}\left(1\right)$ assuming that user *j* keeps its strategy as in frame 0. Each user prefers to jam the other user directly, even if it results in partial decrease of its own s.d.o.f. (by creating an extra dimension at its receiver), since in this case, it can drive the s.d.o.f. of the other user to zero and maximize the s.d.o.f. difference. Thus:

(38) $$\begin{array}{cc} X_{1}\left[1\right] & =X_{1}\left[0\right]+\frac{h_{31}h_{22}}{h_{12}h_{21}}U_{11} \end{array}$$

(39) $$\begin{array}{cc} X_{2}\left[1\right] & =X_{2}\left[0\right]+\frac{h_{32}h_{11}}{h_{12}h_{21}}U_{21} \end{array}$$

**Figure 7:**
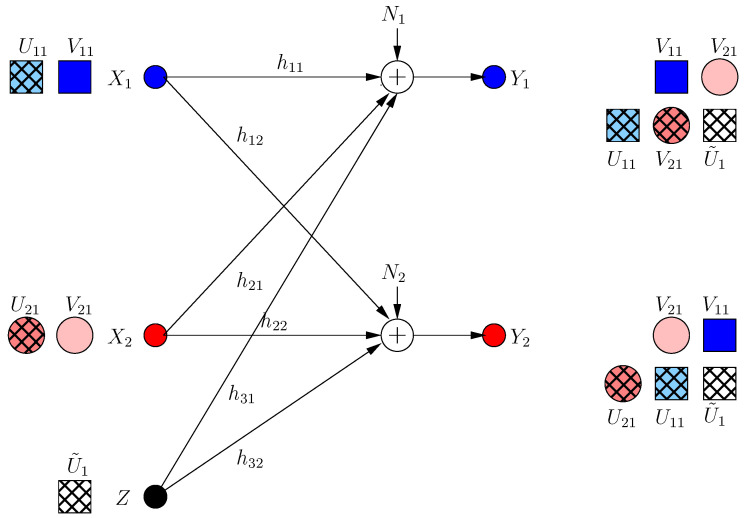
ICCM frame k=1.

Hence, the received signals in this case are:

(40) $$\begin{array}{cc} Y_{1}\left[1\right]= & \frac{h_{32}h_{11}}{h_{12}}\left(V_{11}+U_{21}\right)+h_{31}\left(V_{21}+{\tilde{U}}_{1}\right)+\frac{h_{31}h_{22}h_{11}}{h_{12}h_{21}}U_{11}+N_{1} \end{array}$$

(41) $$\begin{array}{cc} Y_{2}\left[1\right]= & \frac{h_{31}h_{22}}{h_{21}}\left(V_{21}+U_{11}\right)+h_{32}\left(V_{11}+{\tilde{U}}_{1}\right)+\frac{h_{32}h_{12}h_{22}}{h_{12}h_{21}}U_{11}+N_{2} \end{array}$$

which implies that all secure signals are jammed and communication is driven to zero s.d.o.f. as in Figure 7.

#### 3.2.3. For Frame k=2

Both users know that their communication links are jammed during frame k=1. Therefore, the problem of maximizing the s.d.o.f. difference reduces to maximizing s.d.o.f. of each individual user, since the s.d.o.f. of the other user is zero. Each user benefits from the extra jamming dimension created by the other user to protect extra message-carrying component. Moreover, the helper produces an extra jamming component in a new irrational dimension, which allows each user to produce extra secure signal. Thus:

(42) $$\begin{array}{cc} X_{1}\left[2\right] & =X_{1}\left[1\right]+\frac{\alpha_{1}h_{32}}{h_{12}}V_{12}+\frac{h_{32}h_{11}h_{22}}{h_{12}^{2}h_{21}}V_{13} \end{array}$$

(43) $$\begin{array}{cc} X_{2}\left[2\right] & =X_{2}\left[1\right]+\frac{\alpha_{1}h_{31}}{h_{21}}V_{22}+\frac{h_{31}h_{22}h_{11}}{h_{21}^{2}h_{12}}V_{23} \end{array}$$

(44) $$\begin{array}{cc} Z[2] & =Z\left[1\right]+\alpha_{1}{\tilde{U}}_{2} \end{array}$$

where $\alpha_{1}$ is an irrational number independent from all channel gains. Hence, the received signals are:

(45) $$\begin{array}{cc} Y_{1}\left[2\right]= & Y_{1}\left[1\right]\;+\;\alpha_{1}h_{31}\left(V_{22}\;+\;{\tilde{U}}_{2}\right)\;+\;\frac{h_{31}h_{22}h_{11}}{h_{21}h_{12}}V_{23}\;+\;\frac{\alpha_{1}h_{32}h_{11}}{h_{12}}V_{12}\;+\;\frac{h_{32}h_{11}^{2}h_{22}}{h_{12}^{2}h_{21}}V_{13} \end{array}$$

(46) $$\begin{array}{cc} Y_{2}\left[2\right]= & Y_{2}\left[1\right]\;+\;\alpha_{1}h_{32}\left(V_{12}\;+\;{\tilde{U}}_{2}\right)\;+\;\frac{h_{32}h_{11}h_{22}}{h_{12}h_{21}}V_{13}\;+\;\frac{\alpha_{1}h_{31}h_{22}}{h_{21}}V_{22}\;+\;\frac{h_{31}h_{22}h_{11}}{h_{21}^{2}h_{12}}V_{23} \end{array}$$

**Figure 8:**
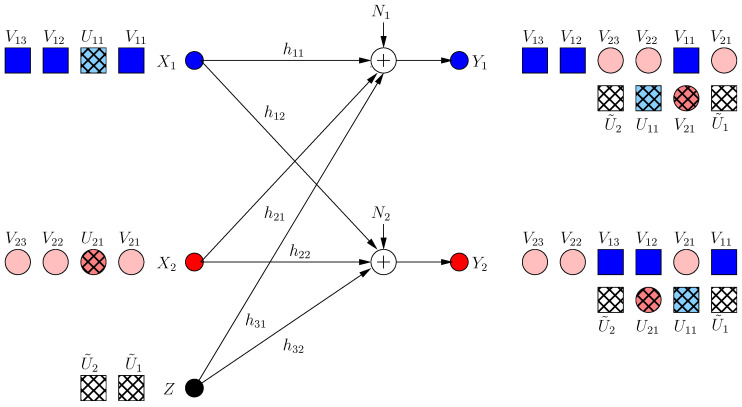
ICCM frame k=2.

Consequently, $d_{i}\left(2\right)=1/3$ as shown in Figure 8.

#### 3.2.4. For General *k*th Frame

The s.d.o.f. differs based on whether *k* is odd/even. If *k* is odd, each user chooses to jam all dimensions of the other user’s secure signals. This choice leads to $d_{i}\left(k\right)=0$ for all odd frames. If *k* is even, each user takes advantage of the generated jamming by the other user plus the extra jamming signal from the system helper to protect more signals.

### 3.3. Calculation of the Secure Degrees of Freedom

**Theorem** **2.** *For the ICCM with selfish users in the presence of a system helper, assuming that users maximize the s.d.o.f. difference for every transmission frame, the s.d.o.f. evolves as:*

(47) $$d_{i}\left(k\right)=\left\{\begin{array}{cc} 0, & k\;odd\\ \frac{2}{k+4}\to 0, & k\;even \end{array}\right.$$

*i.e., selfishness eventually precludes secure communication.*

**Proof.**  From [5], the rates given in (19) are achievable for the ICCM. Then, from Lemma 1, we have $d_{i}\left(k\right)=\frac{J_{k}}{L_{k}}$. Next, we count $J_{k}=\frac{k+2}{2}$ when *k* is even. This follows by induction: For k=1, the number of secure dimensions is 1. Now, assume that the relation holds for any even k−2. Then, $J_{k-2}=\frac{k}{2}$. Then, since user *i* jams all secure dimensions of user *j* in frame k−1, it creates $\frac{k}{2}$ new dimensions. These dimensions are used by user *i* in frame *k* to protect $\frac{k}{2}$ new secure signals. The helper produces an extra jamming component, allowing protection of one extra signal. Then, $J_{k}=\frac{k}{2}+1=\frac{k+2}{2}$.

We use this result in proving s.d.o.f. by induction: For k=0, $J_{0}=1$ and $L_{0}=2$, which leads to $d_{i}\left(0\right)=1/2$. For k=1, $J_{1}=0$ and $L_{1}=3$, which leads to $d_{i}\left(1\right)=0$. Now, assume that *k* is even and expression (47) is true, then, $d_{i}\left(k-2\right)=\frac{2}{k+2}$. Then, from the above, we have $J_{k-2}=\frac{k}{2}$. Hence, $L_{k-2}=\frac{k(k+2)}{4}$. The total dimensions $L_{k}$ at any receiver is increased over the k−2 frame by $2J_{k}$, since the increase is caused by the new secure dimensions $J_{k}$ for the two users, which are symmetric. Therefore, the s.d.o.f. for even *k* is:

(48) $$\begin{array}{c} d_{i}\left(k\right)=\frac{J_{k}}{L_{k}}=\frac{J_{k}}{L_{k-2}+2J_{k}}=\frac{2}{k+4} \end{array}$$

If *k* is odd, users make s.d.o.f. zero, completing the proof. □

**Remark** **1.** *Although the previous channel models are different, they have critical similarities: In both models, there is a central node, transmitter in BCCM, and helper in ICCM, which altruistically want to maximize the sum s.d.o.f.; however, the transmitter in BCCM can send useful signals, but the helper ICCM can only jam. In both models, there are two adversarial/selfish transmitters, helpers in BCCM, and users in ICCM; however, helpers in BCCM can only jam, but users in ICCM can send useful signals and/or jam. We observe that this difference in roles drives systems to opposite end results of full s.d.o.f. in BCCM and zero s.d.o.f. in ICCM.*

## 4. Multiple Access Wiretap Channel with Deviating Users

### 4.1. System Model and Assumptions

**Figure 9:**
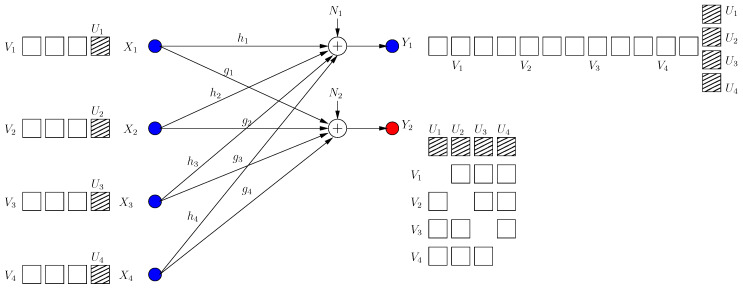
Optimal achievable scheme for a K=4 user multiple access wiretap channel (MAC-WTC).

The *K*-user Gaussian MAC-WTC is given by (see Figure 9):

(49) $$\begin{array}{cc} Y_{1} & =\sum_{i=1}^{K}h_{i}X_{i}+N_{1} \end{array}$$

(50) $$\begin{array}{cc} Y_{2} & =\sum_{i=1}^{K}g_{i}X_{i}+N_{2} \end{array}$$

where $Y_{1},Y_{2}$ are the channel outputs at the legitimate receiver and the eavesdropper, respectively, and $h_{i},g_{i}$ are the channel gains from user *i* to the receiver and the eavesdropper, respectively. User *i* has a message $W_{i}$ picked uniformly from the message set $W_{i}$, with a rate $R_{i}=\frac{1}{n}\log\left|W_{i}\right|$, and sends it in *n* channel uses using $X_{i}^{n}$ reliably and securely, i.e.,

(51) $$\begin{array}{c} P\left({\hat{W}}_{1}^{K}\neq W_{1}^{K}\right)\leq \epsilon ,\;\frac{1}{n}I\left(W_{1}^{K};Y_{2}^{n}\right)\leq \epsilon \end{array}$$

where $W_{1}^{K}=\left(W_{1},\cdots ,W_{K}\right)$, and ${\hat{W}}_{1}^{K}=\left({\hat{W}}_{1},\cdots ,{\hat{W}}_{K}\right)$ are the estimates of the messages at the legitimate receiver. The transmitters are subject to power constraints $E[X_{i}^{2}]\leq P$. The sum s.d.o.f. is given by $d_{s}=\lim_{P\to \infty }\frac{\sum_{i=1}^{K}R_{i}}{\frac{1}{2}\log P}$.

In the second part of the section, we consider a severe form of deviation where one user transmits intentional jamming signals. To distinguish that user and its jamming signal, we denote its channel input as *Z*, which is also subject to the power constraint $E[Z^{2}]\leq P$, and we designate it as the *K*th user without loss of generality, see Figure 13. The malicious user and the remaining users respond to each other in multiple coding frames. The channel inputs/outputs for this model in frame *k* are:

(52) $$\begin{array}{cc} Y_{1}\left[k\right] & =\sum_{i=1}^{K-1}h_{i}X_{i}\left[k\right]+\tilde{h}Z\left[k\right]+N_{1}\left[k\right] \end{array}$$

(53) $$\begin{array}{cc} Y_{2}\left[k\right] & =\sum_{i=1}^{K-1}g_{i}X_{i}\left[k\right]+\tilde{g}Z\left[k\right]+N_{2}\left[k\right] \end{array}$$

where $\tilde{h},\tilde{g}$ are the channel gains from the malicious user to the legitimate receiver and the eavesdropper, respectively.

### 4.2. S.d.o.f. When Remaining Users Do Not Respond

**Figure 10:**
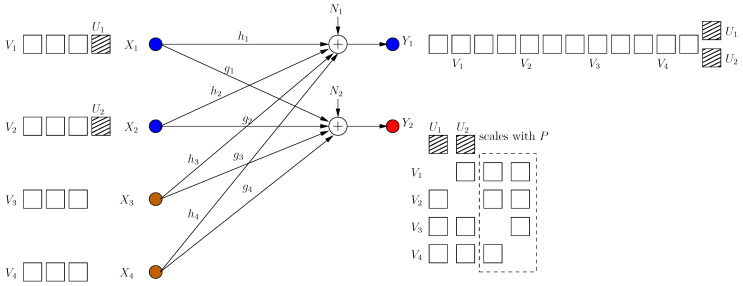
The remaining users keep their originally optimum schemes.

Consider that *M* users have deviated from the optimum strategy in [13] (see Figure 9) by not sending cooperative jamming signals and that the remaining users have kept their originally optimum strategies, i.e., have not responded to the deviating users (see Figure 10). That is, the user signals are [13]:

(54) $$X_{i}=\left\{\begin{array}{cc} \sum_{j=1,j\neq i}^{K}\frac{g_{j}}{g_{i}h_{j}}V_{ij}+\frac{1}{h_{i}}U_{i}, & \;i=1,\cdots ,K-M\\ \sum_{j=1,j\neq i}^{K}\frac{g_{j}}{g_{i}h_{j}}V_{ij}, & \;i=K-M+1,\cdots ,K \end{array}\right.$$

where $V_{ij},U_{i}$ are picked uniformly from PAM constellation set C(a,Q) [13]. The constants a,Q are chosen as [13]:

(55) $$\begin{array}{c} Q=P^{\frac{1-\delta }{2K(K-1)+1+\delta }},\;a=\gamma \frac{P^{1/2}}{Q} \end{array}$$

Consequently, the received signals are (see Figure 10):

(56) $$\begin{array}{cc} Y_{1}= & \sum_{i=1}^{K}\sum_{j=1,j\neq i}^{K}\frac{g_{j}h_{i}}{g_{j}h_{j}}V_{ij}+\sum_{k=1}^{K-M}U_{k}+N_{1} \end{array}$$

(57) $$\begin{array}{cc} Y_{2}= & \sum_{i=1}^{K}\sum_{j=1,j\neq i}^{K}\frac{g_{j}}{h_{j}}V_{ij}+\sum_{j=1}^{K-M}\frac{g_{j}}{h_{j}}U_{j}+N_{2} \end{array}$$

(58) $$\begin{array}{cc} = & \sum_{j=1}^{K-M}\frac{g_{j}}{h_{j}}\left(U_{j}+\sum_{i=1,i\neq j}^{K}V_{ij}\right)+\sum_{j=K-M+1}^{K}\sum_{i=1,i\neq j}^{K}\frac{g_{j}}{h_{j}}V_{ij}+N_{2} \end{array}$$

Let $V=\{V_{ij}:i,j=1,\cdots K,i\neq j\}$. From [13,15], the following secure rates are achievable:

(59) $$\sum_{i=1}^{K}R_{i}\geq I\left(V;Y_{1}\right)-I\left(V;Y_{2}\right)$$

For the first term $I(V;Y_{1})$: We note that the components of vector V are received in different rational dimensions, and hence, we have ${\left(2Q+1\right)}^{K(K-1)}$ separable constellation points, while the cooperative jamming signal components are aligned in the same rational dimension, i.e., (2(K−M)Q+1) constellation points. From data processing and Fano’s inequalities:

(60) $$\begin{array}{cc} I(V;Y_{1}) & \geq I\left(V;\hat{V}\right)=H\left(V\right)-H\left(V|\hat{V}\right) \end{array}$$

(61) $$\begin{array}{cc} \; & \geq \left[1-\exp\left(\eta_{\gamma }P^{\delta }\right)\right]\log{\left(2Q+1\right)}^{K(K-1)}-1 \end{array}$$

(62) $$\begin{array}{cc} \; & =\frac{K(K-1)(1-\delta )}{K(K-1)+1+\delta }\cdot \frac{1}{2}\log P+o\left(\log P\right) \end{array}$$

For the second term $I(V;Y_{2})$: We note that we have K−M dimensions, in which message-carrying signals are aligned with cooperative jamming signals, while *M* dimensions lack cooperative jamming signals, i.e., we have ${\left(2KQ+1\right)}^{K-M}\cdot {\left(2\left(K-1\right)Q+1\right)}^{M}$ constellation points. Hence:

(63) $$\begin{array}{cc} I(V;Y_{2})\leq & H\left(Y_{2}-N_{2}\right)-H\left(Y_{2}-N_{2}|V\right) \end{array}$$

(64) $$\begin{array}{cc} \leq & \log{\left(2KQ+1\right)}^{K-M}{\left(2\left(K-1\right)Q+1\right)}^{M}{)-\log\left(2Q+1\right)}^{K-M} \end{array}$$

(65) $$\begin{array}{cc} = & \left(K-M\right)\log\frac{2KQ+1}{2Q+1}+M\log\left(2\left(K-1\right)Q+1\right) \end{array}$$

(66) $$\begin{array}{cc} \leq & \left(K-M\right)\log K+\frac{M(1-\delta )}{K(K-1)+1+\delta }\cdot \frac{1}{2}\log P+o\left(\log P\right) \end{array}$$

(67) $$\begin{array}{cc} = & \frac{M(1-\delta )}{K(K-1)+1+\delta }\cdot \frac{1}{2}\log P+o\left(\log P\right) \end{array}$$

Substituting (62) and (67) into (59), and taking the limit as P→∞, the achievable sum s.d.o.f. is:

(68) $$d_{s}\geq \frac{K(K-1)-M}{K(K-1)+1}$$

That is, the sum s.d.o.f. decreases by $\frac{M}{K(K-1)+1}$ from the optimal in [13]. This affects all users, including the deviating users; hence, they do not benefit from their deviation.

### 4.3. S.d.o.f. When Remaining Users Respond

In this section, we consider two achievable schemes resulting from two different responses of the remaining users.

#### 4.3.1. Reducing the Secure Rate for Zero Leakage Rate

**Figure 11:**
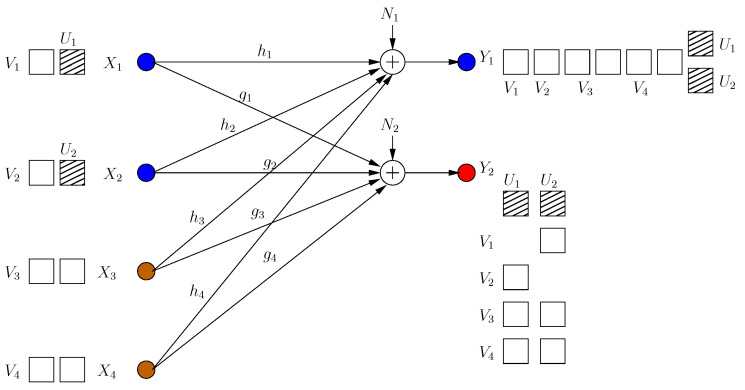
All users reduce rates to have zero leakage secure degrees of freedom (s.d.o.f.)

In this achievable scheme, all users decrease their secure rates, i.e., decrease the number of message-carrying signal components to ensure that all of them are aligned with cooperative jamming signals. Specifically, the first K−M users send K−M−1 message-carrying signals and 1 cooperative jamming signal, while the rest of the users, i.e., the deviating users, send K−M message-carrying signals and no cooperative jamming signals, see Figure 11. Note that the deviating users are motivated to decrease their message-carrying signals from K−1 to K−M, as otherwise, some of their message-carrying signals would not be protected. The transmitted signals are:

(69) $$X_{i}=\left\{\begin{array}{cc} \sum_{j=1,j\neq i}^{K-M}\frac{g_{j}}{g_{i}h_{j}}V_{ij}+\frac{1}{h_{i}}U_{i}, & \;i=1,\cdots ,K-M\\ \sum_{j=1}^{K-M}\frac{g_{j}}{g_{i}h_{j}}V_{ij}, & \;i=K-M+1,\cdots ,K \end{array}\right.$$

Consequently, the received signals are (see Figure 11):

(70) $$\begin{array}{cc} Y_{1}= & \sum_{i=1}^{K-M}\sum_{j=1,j\neq i}^{K-M}\frac{g_{j}h_{i}}{g_{j}h_{j}}V_{ij}+\sum_{i=K-M+1}^{K}\sum_{j=1}^{K-M}\frac{g_{j}h_{i}}{g_{j}h_{j}}V_{ij}+\sum_{k=1}^{K-M}U_{k}+N_{1} \end{array}$$

(71) $$\begin{array}{cc} Y_{2}= & \sum_{i=1}^{K-M}\sum_{j=1,j\neq i}^{K-M}\frac{g_{j}}{h_{j}}V_{ij}+\sum_{i=K-M+1}^{K-M}\sum_{j=1}^{K-M}\frac{g_{j}}{h_{j}}V_{ij}+\sum_{j=1}^{K-M}\frac{g_{j}}{h_{j}}U_{j}+N_{2} \end{array}$$

(72) $$\begin{array}{cc} = & \sum_{j=1}^{K-M}\frac{g_{j}}{h_{j}}\left(U_{j}+\sum_{i=1,i\neq j}^{K-M}V_{ij}+\sum_{i=K-M+1}^{K}V_{ij}\right)+N_{2} \end{array}$$

Let $V=\{V_{ij}:i=1,\cdots K,j=1,\cdots ,K-M,i\neq j\}$. We evaluate the secrecy rates using (59), after choosing:

(73) $$\begin{array}{c} Q=P^{\frac{1-\delta }{2(K-M)(K-1)+1+\delta }},\;a=\gamma \frac{P^{1/2}}{Q} \end{array}$$

The components of V are received in different dimensions, and hence, we have ${\left(2Q+1\right)}^{\left(K-M)(K-M-1)+M(K-M\right)}={\left(2Q+1\right)}^{\left(K-M)(K-1\right)}$ separable constellation points, while the cooperative jamming signals are aligned in the same dimension, i.e., (2(K−M)Q+1) constellation points. Thus:

(74) $$\begin{array}{cc} I(V;Y_{1}) & \geq I(V;\hat{V}) \end{array}$$

(75) $$\begin{array}{cc} \; & =\frac{\left(K-M)(K-1)(1-\delta \right)}{(K-M)(K-1)+1+\delta }\cdot \frac{1}{2}\log P+o\left(\log P\right) \end{array}$$

Since all message-carrying signals are jammed by cooperative jamming signals, we have K−M dimensions with ${\left(2KQ+1\right)}^{\left(K-M\right)}$ overlapping constellation points. Thus:

(76) $$\begin{array}{cc} I(V;Y_{2})\leq & H\left(Y_{2}-N_{2}\right)-H\left(Y_{2}-N_{2}|V\right) \end{array}$$

(77) $$\begin{array}{cc} = & H\left(\sum_{j=1}^{K-M}\frac{g_{j}}{h_{j}}\left(U_{j}+\sum_{i=1,i\neq j}^{K-M}V_{ij}+\;\;\;\sum_{i=K-M+1}^{K}\;\;\;V_{ij}\right)\right)-H\left(\sum_{j=1}^{K-M}\frac{g_{j}}{h_{j}}U_{j}\right) \end{array}$$

(78) $$\begin{array}{cc} = & \left(K-M\right)\log\frac{2KQ+1}{2Q+1} \end{array}$$

(79) $$\begin{array}{cc} \leq & (K-M)\log K \end{array}$$

Substituting (75) and (79) into (59), and taking the limit as P→∞, the achievable sum s.d.o.f. is:

(80) $$d_{s}\geq \frac{\left(K-M)(K-1\right)}{(K-M)(K-1)+1}$$

The resultant sum s.d.o.f. is less than the optimal in [13]. However, interestingly, the individual s.d.o.f. of each deviating user is $\frac{K-M}{(K-M)(K-1)+1}$, which is larger than its s.d.o.f. without deviation $\frac{K-1}{K(K-1)+1}$, so long as $M\leq K-1+\frac{1}{K}$, i.e., if at least one user sticks to the optimal strategy in [13].

#### 4.3.2. Reducing the Leakage to a Single Dimension

**Figure 12:**
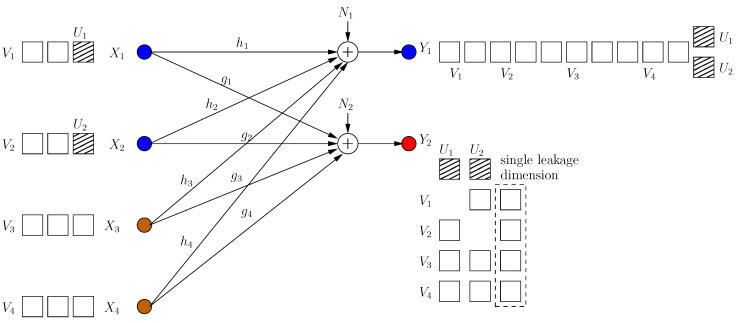
All users reduce the leakage dimension to 1.

In this achievable scheme, we allow one rational dimension to be leaked. This dimension is not secured by a cooperative jamming signal. This results in the ability of injecting an extra message-carrying signal component for each user. All these extra signals are aligned in the same rational dimension at the eavesdropper. The transmitted signals are (see Figure 12):

(81) $$X_{i}=\left\{\;\;\begin{array}{c} \sum_{j=1,j\neq i}^{K-M}\frac{g_{j}}{g_{i}h_{j}}V_{ij}+\frac{\alpha }{h_{i}}V_{i0}+\frac{1}{h_{i}}U_{i},i=1,\cdots ,K\;-\;M\\ \sum_{j=1}^{K-M}\frac{g_{j}}{g_{i}h_{j}}V_{ij}+\frac{\alpha }{h_{i}}V_{i0},\;i=K-M+1,\cdots ,K \end{array}\right.$$

where α is rationally independent from all channel gains. The received signals are shown in Figure 12. Through similar steps, we have the following s.d.o.f. for this scheme:

(82) $$d_{s}\geq \frac{{\left(K-M\right)}^{2}+M\left(K-M+1\right)-1}{{\left(K-M\right)}^{2}+M\left(K-M+1\right)+1}$$

Although the sum s.d.o.f. in this case is smaller than in (80), the individual s.d.o.f. of a well-behaving user is higher and a deviating user is lower than in (80).

### 4.4. Malicious Deviation: Intentional Jamming

In this section, we consider a more severe form of deviation, where a user (say the *K*th user) sends intentional jamming signals. The deviating (malicious) user is restricted to using structured signals. In this section, we show that, when the malicious user acts, it can drive the sum s.d.o.f. to zero. However, when the remaining users respond, the sum s.d.o.f. is raised to $d_{s}=\frac{{\left(K-1\right)}^{2}}{{\left(K-1\right)}^{2}+1}$, which is the sum s.d.o.f. of a K−1 user MAC-WTC with an external altruistic helper.

#### 4.4.1. When the Jammer Responds to the Users

In any encoding frame, each user sends its message-carrying signals $V_{ij}$ on *N* rationally independent dimensions $\alpha_{ij}$ as:

(83) $$X_{i}\left[k\right]=\sum_{j=1}^{N}\alpha_{ij}V_{ij}$$

Then, the jammer designs structured jamming signals ${\tilde{U}}_{ij}$ as a response to users’ signals as:

(84) $$Z\left[k\right]=\sum_{i=1}^{K-1}\sum_{j=1}^{N}\frac{\alpha_{ij}h_{i}}{\tilde{h}}{\tilde{U}}_{ij}$$

Consequently, the received signal at the legitimate receiver is:

(85) $$Y_{1}\left[k\right]=\sum_{i=1}^{K-1}\sum_{j=1}^{N}h_{i}\alpha_{ij}\left(V_{ij}+{\tilde{U}}_{ij}\right)+N_{1}\left[k\right]$$

Hence, each message-carrying signal is aligned with a jamming signal. Let $V\left[k\right]={\left[V_{ij},i=1,\cdots ,K-1,j=1,\cdots ,N\right]}^{T}$ to be a vectorization of all secure signal components. Then, the secure rate is upper bounded as:

(86) $$\begin{array}{cc} \sum_{i=1}^{K-1}R_{i} & \leq I(V\left[k\right];Y_{1}\left[k\right]-N_{1}\left[k\right]) \end{array}$$

(87) $$\begin{array}{cc} \; & =\sum_{i=1}^{K-1}\sum_{j=1}^{N}H\left(V_{ij}+{\tilde{U}}_{ij}\right)-H\left({\tilde{U}}_{ij}\right) \end{array}$$

(88) $$\begin{array}{cc} \; & \leq \sum_{i=1}^{K-1}\sum_{j=1}^{N}\log\left(4Q+1\right)-\log\left(2Q+1\right) \end{array}$$

(89) $$\begin{array}{cc} \; & \leq N(K-1)=o(\log P) \end{array}$$

Hence, $d_{s}=0$, i.e., whenever the jammer knows the signaling scheme of the users, it nulls the communication by jamming.

#### 4.4.2. When the Users Respond to the Jammer

Since structured jamming signaling suffices to jam the system, the jammer sends structured signals in *N* dimensions:

(90) $$Z\left[k\right]=\sum_{j=1}^{N}\alpha_{j}{\tilde{U}}_{j}$$

**Figure 13:**
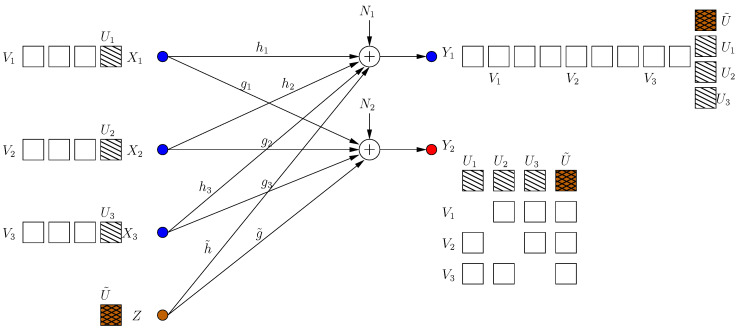
A malicious jamming user: users’ response.

Users make use of the generated jamming signals to hide extra secure signals from the eavesdropper. Users send:

(91) $$X_{i}\left[k\right]=\sum_{j=1}^{N}\sum_{l=1,l\neq i}^{K-1}\frac{\alpha_{j}\tilde{h}g_{l}}{g_{i}h_{i}}V_{ijl}+\sum_{j=1}^{N}\frac{\alpha_{j}\tilde{g}}{g_{i}}V_{ij0}+\sum_{j=1}^{N}\frac{\alpha_{j}\tilde{h}}{h_{i}}U_{ij}$$

where $V_{ijl},V_{ij0}$ are the message-carrying signals which are protected by cooperative jamming signals generated by other users, and the jamming signals generated by the malicious user, respectively. Then, the received signal at receiver 1 is:

(92) $$\begin{array}{cc} Y_{1}\left[k\right]= & \sum_{j=1}^{N}\left(\sum_{i=1}^{K-1}\sum_{l=1,l\neq i}^{K-1}\frac{\alpha_{j}\tilde{h}g_{l}h_{i}}{g_{i}}V_{ijl}+\sum_{i=1}^{K-1}\frac{\alpha_{j}\tilde{g}h_{i}}{g_{i}}V_{ij0}+\alpha_{j}\tilde{h}\left({\tilde{U}}_{j}+\sum_{i=1}^{K-1}U_{ij}\right)\right)+N_{1} \end{array}$$

i.e., users’ jamming signals use the same dimensions as the external jammer to inject extra cooperative jamming signals. The received signal at the eavesdropper is:

(93) $$\begin{array}{cc} Y_{2}\left[k\right]= & \sum_{i=1}^{K-1}g_{i}\left[\sum_{j=1}^{N}\sum_{l=1,l\neq i}^{K-1}\frac{\alpha_{j}\tilde{h}g_{l}}{g_{i}h_{i}}V_{ijl}+\sum_{j=1}^{N}\frac{\alpha_{j}\tilde{g}}{g_{i}}V_{ij0}+\sum_{j=1}^{N}\frac{\alpha_{j}\tilde{h}}{h_{i}}U_{ij}\right]+\tilde{g}\sum_{j=1}^{N}\alpha_{j}{\tilde{U}}_{j}+N_{2} \end{array}$$

(94) $$\begin{array}{cc} = & \sum_{j=1}^{N}\left[\alpha_{j}\tilde{g}\left(\sum_{i=1}^{K-1}V_{ij0}+{\tilde{U}}_{j}\right)+\sum_{l=1}^{K-1}\frac{\alpha_{j}\tilde{h}g_{l}}{h_{l}}\left(U_{ij}+\sum_{i=1,i\neq l}^{K-1}V_{lji}\right)\right]+N_{2} \end{array}$$

i.e., all message-carrying signals are protected from the eavesdropper, as in Figure 13, with K=4, N=1.

We note that the received signals at receiver $Y_{1}$ consist of ${\left(2Q+1\right)}^{N(K-1)(K-2)+N(K-1)}\left(2NKQ+1\right)$ constellation points in $N({\left(K-1\right)}^{2}+1)$ dimensions. Each user is transmitting using PAM constellation C(a,Q). By choosing $Q=P^{\frac{1-\delta }{2N({\left(K-1\right)}^{2}+1)+\delta }}$ and $a=\gamma P^{\frac{1}{2}}/Q$, we have:

(95) $$\begin{array}{cc} I(V;Y_{1}\left[k\right]) & \geq \frac{N{\left(K-1\right)}^{2}\left(1-\delta \right)}{N({\left(K-1\right)}^{2}+1)+\delta }\left(\frac{1}{2}\log P\right)+o\left(\log P\right) \end{array}$$

Further, since every message-carrying signal is protected by a cooperative jamming signal, $I(V;Y_{2}\left[k\right])\leq o\left(\log P\right)$. Thus, the achievable sum s.d.o.f. with one malicious jammer when users respond is $d_{s}\left(k\right)=\frac{{\left(K-1\right)}^{2}}{{\left(K-1\right)}^{2}+1}$. Finally, in the Appendix A, we determine the sum s.d.o.f. of a *K*-user MAC-WTC with *M* external altruistic helpers, as a result on its own. We note that this $d_{s}\left(k\right)$ is in fact equal to the sum s.d.o.f. of a K−1 user MAC-WTC with one external helper, concluding that the users’ action to the jammer is optimal, as they achieve the s.d.o.f. of the case of an altruistic helper with a malicious jammer.

## 5. Conclusions

We introduced three new channel models, namely, BCCM with combating helpers, ICCM with selfish users, and MAC-WTC with deviating users. These new models aimed at studying the effects of selfishness and malicious behavior on the secure rate in networks. We investigated the achievable s.d.o.f. in these models. The presented schemes are only achievable; new role-based converse arguments are needed.

For the BCCM with combating helpers, we formulated the problem as an extensive-form game. We assumed that each helper wants to minimize the s.d.o.f. of the other receiver without sacrificing the s.d.o.f. of its receiver and analyzed schemes that employ recursive real interference alignment. In this case, we showed that the malicious behaviors of the combating helpers are neutralized and the s.d.o.f. of both users converge to 1/2, as in the case of altruistic helpers.

For the ICCM with selfish users, we changed the objective function of the users to maximizing the difference of the s.d.o.f. between the two users. By similar analysis to BCCM, we showed that the selfishness precludes any secure communication, and the s.d.o.f. of two users converge to zero.

Finally, for the MAC-WTC with deviating users, we considered two types of deviation: First, in the case when some of the users stop transmitting cooperative jamming signals as required by the optimal scheme, we evaluated the corresponding s.d.o.f. and proposed counterstrategies to respond to the deviation. Second, we investigated an extreme form of deviation, where a user sends intentional jamming signals. We showed that although a deviating user can drive the sum s.d.o.f. to zero, the jamming signals can be exploited as cooperative jamming signals against the eavesdropper to achieve an optimum s.d.o.f.

## Appendix A. K-User MAC-WTC with M External Helpers

In this Appendix, we present the *exact* s.d.o.f. for a MAC-WTC with *K* users in the presence of *M* external helpers. In the context of user-misbehavior, the s.d.o.f. of this model serves as an upper bound for the MAC-WTC when the users respond to the intentional jamming when the number of users is K−1 and the number of helpers is 1, i.e., we upper bound the s.d.o.f. in this case by replacing the jammer by an altruistic helper.

**Theorem** **A1.**
*The s.d.o.f. of the K-user Gaussian MAC-WTC with M-external helpers is given by $d_{s}=\frac{K(K+M-1)}{K(K+M-1)+1}$.*

**Proof.** For the achievability, each user sends K+M−1 message-carrying signals and one cooperative jamming signal to secure the other users. Each helper sends one cooperative jamming signal. The cooperative jamming signals are aligned in the same rational dimension at the receiver. For the converse, we rely on the techniques in [13]. The received signals at legitimate and eavesdropper receivers of the *K*-user Gaussian MAC with *M* external helpers are given by:

(A1) $$\begin{array}{cc} Y_{1} & =\sum_{i=1}^{K}h_{i}X_{i}+\sum_{j=1}^{M}{\tilde{h}}_{j}Z_{j}+N_{1} \end{array}$$

(A2) $$\begin{array}{cc} Y_{2} & =\sum_{i=1}^{K}g_{i}X_{i}+\sum_{j=1}^{M}{\tilde{g}}_{j}Z_{j}+N_{2} \end{array}$$

where $h_{i},g_{i}$ are the channel gains from the *i*th user to the legitimate receiver and the eavesdropper, respectively, and ${\tilde{h}}_{j},{\tilde{g}}_{j}$ are channel gains from the *j*th helper to the legitimate receiver and the eavesdropper, respectively. $X_{i},Z_{j}$ are input signals from the *i*th user and the *j*th helper, respectively. We denote all *n*-lettered signals by bold vector notation, e.g., let $X_{i}^{n}$ be expressed as $X_{i}$. First, we have the following upper bound which represents the *secrecy penalty* due to the secrecy constraint on the eavesdropper:

(A3) $$\begin{array}{cc} n\sum_{i=1}^{K}R_{i} & =\sum_{i=1}^{K}H\left(W_{i}\right) \end{array}$$

(A4) $$\begin{array}{cc} \; & =H(W_{1}^{K}) \end{array}$$

(A5) $$\begin{array}{cc} & \leq I\left(W_{1}^{K};Y_{1}\right)+H\left(W_{1}^{K}|Y_{1}\right)-I\left(W_{1}^{K};Y_{2}\right)+nc_{1} \end{array}$$

(A6) $$\begin{array}{cc} & \leq I\left(W_{1}^{K};Y_{1},Y_{2}\right)-I\left(W_{1}^{K};Y_{2}\right)+nc_{2} \end{array}$$

(A7) $$\begin{array}{cc} \; & =I\left(W_{1}^{K};Y_{1}|Y_{2}\right)+nc_{2} \end{array}$$

(A8) $$\begin{array}{cc} \; & \leq I\left(X_{1}^{K};Y_{1}|Y_{2}\right)+nc_{2} \end{array}$$

(A9) $$\begin{array}{cc} \; & =h\left(Y_{1}|Y_{2}\right)-h\left(Y_{1}|Y_{2},X_{1}^{K}\right)+nc_{2} \end{array}$$

(A10) $$\begin{array}{cc} & \leq h\left(Y_{1}|Y_{2}\right)-h\left(Y_{1}|Y_{2},X_{1}^{K},Z_{1}^{M}\right)+nc_{2} \end{array}$$

(A11) $$\begin{array}{cc} \; & =h\left(Y_{1}|Y_{2}\right)-h\left(N_{1}\right)+nc_{2} \end{array}$$

(A12) $$\begin{array}{cc} \; & \leq h\left(Y_{1}|Y_{2}\right)+nc_{3} \end{array}$$

(A13) $$\begin{array}{cc} \; & =h\left(Y_{1},Y_{2}\right)-h\left(Y_{2}\right)+nc_{3} \end{array}$$

where $W_{1}^{K}={\left\{W_{i}\right\}}_{1}^{K}$ corresponds to messages 1 through *K*, and similarly, $X_{1}^{K},Z_{1}^{M}$ represent input signals 1 through *K* from the users and input signals 1 through *M* from the helpers, respectively. Inequality (A5) follows from applying the secrecy constraint, (A6) follows from Fano’s inequality due to reliability requirements, and (A10) follows from conditioning over helpers’ input signals. We define Gaussian-perturbed input signals for the users and the helpers to not deal with mixed probability measures as ${\tilde{X}}_{i}=X_{i}+{\tilde{N}}_{i}$, where ${\tilde{N}}_{i}$ is i.i.d. Gaussian noise with variance $\sigma_{i}^{2}<\min\left\{\frac{1}{h_{i}^{2}},\frac{1}{g_{i}^{2}}\right\}$ and similarly, for ${\tilde{Z}}_{i}=Z_{i}+{\bar{N}}_{i}$. We introduce these channel inputs in our bound as:

(A14) $$\begin{array}{cc} n\sum_{i=1}^{K}R_{i} & \leq h\left({\tilde{X}}_{1}^{K},{\tilde{Z}}_{1}^{M},Y_{1},Y_{2}\right)-h\left({\tilde{X}}_{1}^{K},{\tilde{Z}}_{1}^{M}|Y_{1},Y_{2}\right)-h\left(Y_{2}\right)+nc_{3} \end{array}$$

(A15) $$\begin{array}{cc} \; & \leq h\left({\tilde{X}}_{1}^{K},{\tilde{Z}}_{1}^{M},Y_{1},Y_{2}\right)-h\left({\tilde{X}}_{1}^{K},{\tilde{Z}}_{1}^{M}|Y_{1},Y_{2},X_{1}^{K},Z_{1}^{M}\right)-h\left(Y_{2}\right)+nc_{3} \end{array}$$

(A16) $$\begin{array}{cc} & \leq \sum_{i=1}^{K}h\left({\tilde{X}}_{i}\right)+\sum_{j=1}^{M}h\left({\tilde{Z}}_{j}\right)-h\left(Y_{2}\right)+nc_{4} \end{array}$$

where (A16) follows from the fact that ${\tilde{X}}_{1}^{K},{\tilde{Z}}_{1}^{M}$ are reconstructable up to finite variance Gaussian noise given $Y_{1},Y_{2},X_{1}^{K},Z_{1}^{M}$ and applying the independence upper bound for the first term. The differential entropy of the received observations at the eavesdropper can be lower bounded as:

(A17) $$\begin{array}{cc} h(Y_{2}) & =h(g_{j}{\tilde{X}}_{j}+\sum_{i=1,i\neq j}g_{i}X_{i}+\sum_{l=1}^{M}{\tilde{g}}_{l}{\tilde{Z}}_{l}+{\overset{‘}{N}}_{2}) \end{array}$$

(A18) $$\begin{array}{cc} \; & \geq h(g_{j}{\tilde{X}}_{j}) \end{array}$$

(A19) $$\begin{array}{cc} \; & =h\left({\tilde{X}}_{j}\right)+n\log\left|g_{j}\right| \end{array}$$

for some j∈{1,⋯,K}. Consequently, we can write (A16) as the following upper bound which represents the secrecy penalty due to the secrecy constraint imposed on $Y_{2}$

(A20) $$n\sum_{i=1}^{K}R_{i}\leq \sum_{i=2}^{K}h\left({\tilde{X}}_{i}\right)+\sum_{j=1}^{M}h\left({\tilde{Z}}_{j}\right)+nc_{5}$$

Next, we have the *role of the external helper(s)*, i.e., upper bounding the differential entropy of the external helpers to ensure decodability of all messages at the legitimate receiver. The sum rate is upper bounded as:

(A21) $$\begin{array}{cc} n\sum_{i=1}^{K}R_{i} & =H(W_{1}^{K}) \end{array}$$

(A22) $$\begin{array}{cc} & \leq I\left(W_{1}^{K};Y_{1}\right)+nc_{6} \end{array}$$

(A23) $$\begin{array}{cc} & \leq I\left(\sum_{i=1}^{K}h_{i}X_{i};Y_{1}\right)+nc_{6} \end{array}$$

(A24) $$\begin{array}{cc} \; & =h\left(Y_{1}\right)-h\left(Y_{1}|\sum_{i=1}^{K}h_{i}X_{i}\right)+nc_{6} \end{array}$$

(A25) $$\begin{array}{cc} \; & \leq h\left(Y_{1}\right)-h\left(Y_{1}|\sum_{i=1}^{K}h_{i}X_{i},\sum_{l\neq j}{\tilde{h}}_{l}{\tilde{Z}}_{l}\right)+nc_{6} \end{array}$$

(A26) $$\begin{array}{cc} \; & =h\left(Y_{1}\right)-h\left({\tilde{Z}}_{j}\right)+nc_{7} \end{array}$$

where (A30) follows from Fano’s inequality and (A31) follows from the data processing inequality. Therefore, we can upper bound the differential entropy of the *j*th external helper as:

(A27) $$h\left({\tilde{Z}}_{j}\right)\leq h\left(Y_{1}\right)-n\sum_{i=1}^{K}R_{i}+nc_{7}$$

The above argument holds for every external helper, i.e., ∀j∈{1,⋯,M}. By adding the corresponding upper bounds of the *M* helpers, we have the following role of the external helpers upper bound:

(A28) $$\sum_{j=1}^{M}h\left({\tilde{Z}}_{j}\right)\leq Mh\left(Y_{1}\right)-nM\sum_{i=1}^{K}R_{i}+nc_{8}$$

Next, by considering the rates of all users except one for the K−1 users, we have the *role of the internal helper(s)*. Since each user affects the decodability of other users, by upper bounding the message entropy of all users except one, we can obtain an upper bound on the differential entropy of the signaling scheme employed by each user. Let $W_{\neq l}$ be all messages from all users except user *l*:

(A29) $$\begin{array}{cc} n\sum_{i\neq l}R_{i} & =H(W_{\neq l}) \end{array}$$

(A30) $$\begin{array}{cc} & \leq I\left(W_{\neq l};Y_{1}\right)+nc_{9} \end{array}$$

(A31) $$\begin{array}{cc} & \leq I\left(\sum_{i\neq l}h_{i}X_{i};Y_{1}\right)+nc_{9} \end{array}$$

(A32) $$\begin{array}{cc} \; & =h\left(Y_{1}\right)-h\left(Y_{1}|\sum_{i\neq l}h_{i}X_{i}\right)+nc_{9} \end{array}$$

(A33) $$\begin{array}{cc} \; & \leq h\left(Y_{1}\right)-h\left(Y_{1}|\sum_{i\neq l}h_{i}X_{i},\sum_{j=1}^{M}{\tilde{h}}_{l}{\tilde{Z}}_{l}\right)+nc_{9} \end{array}$$

(A34) $$\begin{array}{cc} \; & =h\left(Y_{1}\right)-h\left({\tilde{X}}_{l}\right)+nc_{9} \end{array}$$

Hence, we have the following upper bound on the differential entropy of each user:

(A35) $$h\left({\tilde{X}}_{l}\right)\leq h\left(Y_{1}\right)-n\sum_{i\neq l}^{K}R_{i}+nc_{9}$$

Applying the above upper bound for the K−1 users starting from user 2 to user *K*, we have the following role of the users upper bound:

(A36) $$\sum_{j=2}^{K}h\left({\tilde{X}}_{l}\right)\leq \left(K-1\right)h\left(Y_{1}\right)-n\sum_{l=2}^{K}\sum_{i\neq l}R_{i}$$

Now, we combine all these bounds together. From the upper bounds (A28) and (A36), we substitute in (A20) to have:

(A37) $$\begin{array}{cc} n\sum_{i=1}^{K}R_{i} & \leq \left(K-1\right)h\left(Y_{1}\right)-n\sum_{l=2}^{K}\sum_{i\neq l}R_{i}+Mh\left(Y_{1}\right)-nM\sum_{i=1}^{K}R_{i}+nc_{10} \end{array}$$

We rearrange and simplify (A37) as:

(A38) $$n\left(R_{1}+\left(M+K-1\right)\sum_{i=1}^{K}R_{i}\right)\leq \left(M+K-1\right)h\left(Y_{1}\right)+nc_{10}$$

Noting that (A20) still holds for penalizing any of the users’ rate, then by changing role of users and adding the *K* bounds, we have:

(A39) $$\begin{array}{cc} n\left(\sum_{i=1}^{K}R_{i}+K\left(K+M-1\right)\sum_{i=1}^{K}R_{i}\right) & =n\left(K\left(K+M-1\right)+1\right)\sum_{i=1}^{K}R_{i} \end{array}$$

(A40) $$\begin{array}{cc} \; & \leq K\left(K+M-1\right)h\left(Y_{1}\right)+nc_{11} \end{array}$$

(A41) $$\begin{array}{cc} \; & \leq K\left(K+M-1\right)\left(\frac{n}{2}\log P\right)+nc_{12} \end{array}$$

where we used the fact that Gaussian maximizes differential entropy under an average power constraint. By normalizing by $\frac{1}{2}\log P$ and taking limits as P→∞, we have the following upper bound on the sum s.d.o.f. for the K-user MAC with *M* external helpers as:

(A42) $$d_{s}\leq \frac{K(K+M-1)}{K(K+M-1)+1}$$

concluding the proof of the theorem. □

Note that this result is related to the s.d.o.f. region result in [17] for the K+M user MAC-WTC, when we focus on the hyperplane corresponding to zero s.d.o.f. for *M* of the users; these *M* users essentially serve as helpers.
